# Synergistic Interactions between Cytokines and AVP at the Blood-CSF Barrier Result in Increased Chemokine Production and Augmented Influx of Leukocytes after Brain Injury

**DOI:** 10.1371/journal.pone.0079328

**Published:** 2013-11-01

**Authors:** Joanna Szmydynger-Chodobska, Jessica R. Gandy, Andrew Varone, Rongzi Shan, Adam Chodobski

**Affiliations:** The Neurotrauma and Brain Barriers Research Laboratory, Department of Emergency Medicine, Alpert Medical School of Brown University, Providence, Rhode Island, United States of America; Universidade de São Paulo, Brazil

## Abstract

Several lines of evidence indicate that the blood-cerebrospinal fluid barrier (BCSFB), which primarily resides in the choroid plexus (CP), plays a significant pathophysiological role not only in neuroinflammatory diseases, such as multiple sclerosis, but also in traumatic brain injury (TBI). Here we investigated how arginine vasopressin (AVP) regulates function of the BCSFB in the context of post-traumatic neuroinflammation. It has previously been shown that AVP exacerbates various forms of brain injury, but the mechanisms underlying this AVP action are poorly understood. Type 1A AVP receptor is highly expressed on the CP epithelium and the CP synthesizes AVP. Using the controlled cortical impact model of TBI, we demonstrated decreased post-traumatic production of proinflammatory mediators by the CP and reduced influx of inflammatory cells across the BCSFB in AVP-deficient Brattleboro rats when compared with Long-Evans rats, a parental strain for Brattleboro rats. Arginine vasopressin was also found to play an important role in post-traumatic activation of c-Jun N-terminal kinase (JNK) in the CP. In the CP epithelial cell cultures, AVP augmented the tumor necrosis factor-α– and interleukin-1β–dependent increase in synthesis of proinflammatory mediators, including neutrophil chemoattractants, an action largely dependent on the JNK signaling pathway. Under *in vivo* conditions, a selective JNK inhibitor decreased the post-traumatic production of neutrophil chemoattractants by the CP and reduced the influx of neutrophils across the BCSFB. These results provide evidence for the synergistic interactions between proinflammatory cytokines and AVP, a ligand for G protein-coupled receptors, and support a pathophysiological role of AVP in post-traumatic neuroinflammation.

## Introduction

The choroid plexus (CP) is best known for its ability to produce cerebrospinal fluid (CSF), but this highly vascularized tissue located in all four cerebral ventricles is also a major site for the blood-CSF barrier (BCSFB). The BCSFB is formed by a single layer of cuboidal epithelial cells enclosing the leaky choroidal microvessels [1]. Several lines of evidence indicate that the BCSFB plays an important pathophysiological role in neuroinflammatory diseases, such as multiple sclerosis [2], [3]. We have also demonstrated that in traumatic brain injury (TBI), which is accompanied by neuroinflammation, the BCSFB plays a significant role in synthesis of proinflammatory cytokines and CXC and CC chemokines, and in post-traumatic invasion of neutrophils and monocytes [4], [5]. Post-traumatic influx of leukocytes represents an integral part of secondary injury processes, and laboratory studies of TBI have shown that invading leukocytes significantly augment the formation of cerebral edema, the loss of neural tissue, and functional deficits resulting from injury [6]–[10]. Since post-traumatic neuroinflammation progresses at a relatively slow pace, there is an extended window of opportunity for anti-inflammatory intervention in TBI [11].

In the present study, we investigated the effect of arginine vasopressin (AVP) on function of the BCSFB. It has been demonstrated that AVP plays an important pathophysiological role in various forms of brain injury, including TBI, cerebral ischemia, and intracerebral hemorrhage [12]–[16], but the underlying molecular and cellular mechanisms are not well understood. These investigations are supported by observations that the type 1A AVP receptor (AVPR1A) is highly expressed on the choroidal epithelium [17] and that the CP has the ability to produce AVP [18]. Here we tested the hypothesis that AVP acts synergistically with proinflammatory cytokines tumor necrosis factor-α (TNF-α) and interleukin-1β (IL-1β) to amplify the brain inflammatory response to injury. Using AVP-deficient Brattleboro rats and their parental Long-Evans strain, we showed that AVP increases the post-traumatic production of proinflammatory mediators by the CP and significantly enhances the leukocyte influx across the BCSFB. We also showed that AVP has a substantial effect on post-traumatic activation of c-Jun N-terminal kinase (JNK) in the choroidal tissue. In the CP epithelial cell cultures, AVP amplified the TNF-α– and IL-1β–dependent synthesis of neutrophil chemoattractants. This AVP action was primarily mediated by the JNK signaling cascade. Wild-type rats treated with a selective JNK inhibitor showed a decrease in post-traumatic production of CXC chemokines in the CP and a reduction in the magnitude of neutrophil influx across the BCSFB.

## Materials and Methods

### Rats

Adult male Long-Evans and homozygous AVP-deficient Brattleboro rats (*Avp* ^di/di^) weighing 200–250 g (Harlan, Indianapolis, IN) were used. Brattleboro rats have a single-nucleotide deletion in the *Avp* gene [19]. The rats were kept at 22°C with a 12-h light cycle and maintained on standard pelleted rat chow and water *ad libitum*.

### Reagents and antibodies

ThermoScript RNase H^–^ reverse transcriptase and RNase inhibitor RNaseOut were obtained from Invitrogen (Carlsbad, CA). HotStart *Taq* DNA polymerase was purchased from Qiagen (Valencia, CA). Recombinant rat TNF-α and IL-1β were obtained from R&D Systems (Minneapolis, MN), whereas synthetic AVP was purchased from Bachem (Torrance, CA). Selective inhibitors of JNK (SP600125), MEK1 (PD-98059), p38 (SB203580), and nuclear factor-κB (NF-κB) (SN50) were obtained from Enzo Life Sciences (Farmingdale, NY). Low-endotoxin bovine serum albumin (BSA; 81-068) was from Millipore (Billerica, MA).

The following rabbit polyclonal antibodies were used: anti-human phosphorylated (p)-JNK1/2 (0.2 µg/ml) from Enzo and anti-human JNK2 (diluted 1∶1000) from Cell Signaling Technology (Danvers, MA). The rabbit monoclonal antibodies were as follows: anti-human c-Jun (clone 60A8; diluted 1∶200) and anti-human activating transcription factor 2 (ATF2) (clone 20F1; diluted 1∶200) both from Cell Signaling. The following mouse monoclonal antibodies were used: anti-human p-c-Jun (sc-822; 1 µg/ml) and p-ATF2 (sc-8398; 1 µg/ml) from Santa Cruz Biotechnology (Santa Cruz, CA), and anti-chicken α-tubulin (clone DM1A; diluted 1∶5000) from Cell Signaling. For detection on Western blots, horseradish peroxidase-conjugated anti-rabbit and anti-mouse antibodies from goat (Cell Signaling) were used diluted 1∶5000.

### The rat model of TBI

The surgical and animal care procedures used in this study were approved by the Animal Care and Use Committee of Rhode Island Hospital and conformed to international guidelines on the ethical use of animals. Six to nine rats per group/time point were used. The controlled cortical impact model of TBI was employed as previously described [4], [5]. In brief, rats were anesthetized with intraperitoneal pentobarbital sodium (60 mg/kg) and a 4-mm craniotomy was performed on the right side of the skull to expose the dura, with the center of the opening located 3 mm posterior to the bregma and 2.5 mm lateral to the midline. The velocity of impact was 5 m/sec and the duration of impact was 50 msec. The diameter of the impactor's tip was 2.5 mm and the depth of brain deformation was set at 3 mm. At selected time points after injury, the rats were perfused transcardially with ice-cold 0.9% NaCl and the samples of the lateral ventricle CP, both ipsilateral and contralateral to injury, were collected separately and pooled into three subgroups (2–3 rats per subgroup). To inhibit the JNK activity in vivo, a selective JNK inhibitor SP600125 was injected i.p. at 4 and 8 h post-TBI at a dose of 30 mg/kg for each injection. SP600125 was dissolved in DMSO at a concentration of 12.5 mg/ml.

### CP epithelial cell cultures

Immortalized rat CP epithelial cells Z310 [20] were kindly provided by Dr. Wei Zheng (Purdue University, West Lafayette, IN). These cells have previously been characterized [21]. Low-glucose Dulbecco's modified Eagle medium supplemented with penicillin (100 U/ml), streptomycin (100 µg/ml), gentamicin (40 µg/ml), and 10% fetal bovine serum was used to culture Z310 cells. They were grown to confluence in six-well cell culture plates at 37°C in a humidified atmosphere of 5% CO_2_/95% air. The cells were serum starved in serum-free medium containing 0.1% BSA for 24 h prior to experimentation. On the day of experiment, the cells were exposed to TNF-α or AVP alone, or to the combination of TNF-α and AVP. A separate series of experiments was also performed in which IL-1β was used instead of TNF-α. To inhibit JNK or p38 activity, or to interfere with extracellular signal-regulated kinase (ERK) or NF-κB signaling, the Z310 cells were pre-incubated for 1 h with respective inhibitors and these inhibitors were also added to the culture media when the cells were exposed to TNF-α and AVP. The concentrations of CXCL1–3 and lipocalin 2 (LCN2) in the culture media were determined by ELISA using the DouSet kits from R&D Systems.

### Real-time RT-PCR

Total RNA was isolated using NucleoSpin RNA II kit (Macherey-Nagel, Düren, Germany). First-strand cDNAs were synthesized using oligo(dT)_20_ primer (50 pmol) and 15 U of ThermoScript RNase H^–^ reverse transcriptase. The 20-µl reverse transcription reactions also contained 40 U of RNase inhibitor RNaseOut. For each reaction, 0.5 or 1 µg of total RNA was used and the reactions were carried out for 1 h at 50°C. Real-time PCR was performed using TaqMan chemistry. The sequences of primers and TaqMan probes for TNF-α, IL-1β, CXCL1–3, and CCL2 were previously described [4], [5]. The following primers and TaqMan probe were used for LCN2: 5′-CTGTACGGAAGAACCAAGGG-3′ (forward primer), 5′-CATTGGTCGGTGGGAACA-3′ (reverse primer), 5′-CTGTCCGATGAACTGAAGGAGCGAT-3′ (probe). Cyclophilin A was used for the normalization of the data obtained [4], [5]. The 25-µl PCR reaction mixtures contained 0.2 mM mixed dNTPs, 0.2 µM each primer, 0.1 µM TaqMan probe, 5 mM MgCl_2_, 1 U of HotStart *Taq* DNA polymerase, and 1/20 of the reverse transcription reaction product. For cyclophilin A, 1/1000 of the reverse transcription reaction product was used. The reaction mixtures were heated to 95°C for 15 min and were then subjected to 45 cycles of denaturation (15 sec) at 94°C and annealing/extension (60°C, 60 sec).

### Western blotting

Proteins from the CP or Z310 cells were extracted using isotonic lysis buffer (150 mM NaCl, 50 mM Tris-HCl, pH 7.4, 2 mM EDTA, 1% Triton X-100), containing protease inhibitors (1 mM benzamidine, 100 U/ml aprotinin, 20 µg/ml antipain, 20 µg/ml leupeptin, 1 µg/ml pepstatin A, 1 mM PMSF) and phosphatase inhibitors (10 mM sodium pyrophosphate, 1 mM sodium orthovanadate, 1 mM sodium fluoride, 1 mM β-glycerophosphate). Proteins were resolved via SDS-polyacrylamide gel (4–12%) electrophoresis under reducing conditions and were transferred onto 0.2-µm nitrocellulose membranes (Invitrogen). After blocking with 5% ECL Advance blocking agent (GE Healthcare, Little Chalfont, UK) for 1 h at room temperature, the membranes were incubated with primary antibodies overnight at 4°C. Membranes were subsequently incubated with horseradish peroxidase-conjugated anti-rabbit or anti-mouse antibody for 1 h at room temperature. For detection, Lumigen TMA-6 (Lumigen, Southfield, MI) or SuperSignal West Dura extended duration (Pierce, Rockford, IL) chemiluminescence substrate and the Bio Imaging System Chemi Genius2 (Syngene, Frederick, MD) were used. In the analysis of the optical density of the bands on immunoblots, the levels of phosphorylated JNK, c-Jun, and ATF2 were normalized to the levels of total phosphorylated and non-phosphorylated forms of these proteins. This analysis was performed using ImageJ software (http://rsb.info.nih.gov/ij/).

### JNK activity assay

Non-radioactive JNK activity assays were performed using recombinant c-Jun as a JNK substrate. The assays were completed in kinase buffer (25 mM Tris-HCl, pH 7.4, 10 mM MgCl_2_, 2 mM DTT), containing 0.1 mM sodium orthovanadate and 5 mM β-glycerophosphate. The 50-µl reaction mixtures contained 10 µg of total protein (extracted either from the CP or Z310 cells), 200 ng of c-Jun fusion protein (Cell Signaling), and 200 µM ATP. The reactions were carried out for 30 min at 30°C. The reactions were terminated with 25 µl of 3X SDS sample buffer and the reaction mixtures were boiled for 5 min. Ten microliters of each reaction mixture were loaded on SDS-polyacrylamide gel, and after electrophoresis, proteins were transferred onto nitrocellulose membranes. The membranes were incubated with anti-p-c-Jun antibody (40 ng/ml). The amount of total c-Jun was assessed using anti-c-Jun antibody diluted 1∶5000.

### Myeloperoxidase (MPO) activity assay

To assess the number of neutrophils accumulating in the ipsilateral CP after trauma, the MPO activity assays were performed as previously described [4]. The activity of MPO was assayed spectrophotometrically at 24 h after TBI. One unit of MPO activity was defined as the degradation of 1 µmol of H_2_O_2_ per min. The results are expressed as units of MPO activity per gram of tissue.

### Statistical analysis

For statistical evaluation of data, ANOVA was used, followed by the Newman-Keuls test for multiple comparisons among means. The results are presented as mean values ± SEM. *p*<0.05 was considered statistically significant.

## Results

### Vasopressin upregulates the post-traumatic production of proinflammatory mediators by the CP

**Figure 1:**
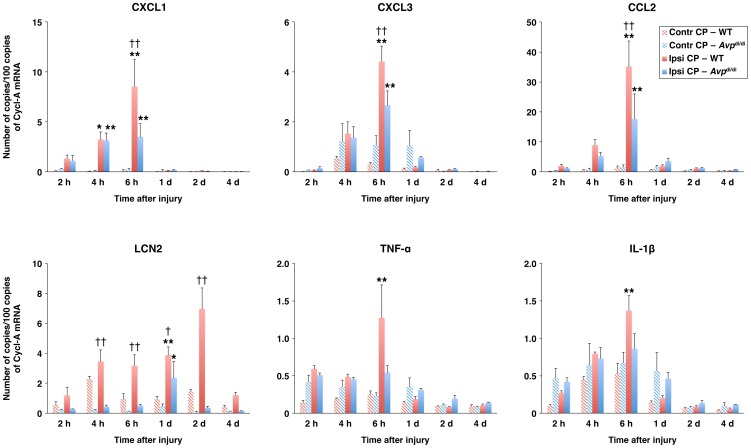
Post-traumatic synthesis of proinflammatory mediators by the lateral ventricle choroid plexus (CP) as assessed by real-time RT-PCR. A comparison of AVP-deficient Brattleboro rats (*Avp* ^di/di^) with their parental Long-Evans strain (WT). The production of proinflammatory mediators in the ipsilateral (Ipsi CP) and contralateral (Contr CP) CPs is shown. Cyclophilin A (Cycl-A) was used for the normalization of the data. *p<0.05, **p<0.01 for the ipsilateral versus contralateral CP. ^†^ *p*<0.05, ^††^ *p*<0.01 for *Avp* ^di/di^ versus WT rats (*n* = 6–9 per rat strain per time point).

Using the same rodent model of TBI, which produces unilateral injury to the cerebral cortex and subcortical structures, we have previously shown that TBI results in a rapid and substantial increase in choroidal production of proinflammatory cytokines TNF-α and IL-1β, and neutrophil and monocyte chemoattractants [4], [5]. To investigate the possible role of AVP in augmenting the post-traumatic production of proinflammatory mediators by the CP, we used the homozygous AVP-deficient Brattleboro rats. When compared with their parental wild-type Long-Evans strain, *Avp* ^di/di^ rats had reduced levels (by 50–59%) of mRNA for CXC and CC chemokines in the ipsilateral CP at 6 h post-TBI, a time point after injury at which a peak in chemokine synthesis was observed in these two rat strains (Fig. 1). Since TNF-α and IL-1β are well-known inducers of chemokine synthesis, we also assessed the differences in post-traumatic production of these two cytokines in *Avp* ^di/di^ versus wild-type rats. The levels of mRNA for TNF-α and IL-1β in the ipsilateral CP at 6 h post-TBI were lower by 57% and 37%, respectively, in *Avp* ^di/di^ versus wild-type rats; however, these differences did not attain statistical significance because of variations in the levels of cytokine synthesis among individual animals. A significant difference between *Avp* ^di/di^ and wild-type rats was observed in post-traumatic production of LCN2, a proinflammatory mediator whose synthesis in the CP epithelium is highly upregulated in response to inflammatory stimuli [22]. Unlike the chemokine synthesis, the post-traumatic production of LCN2 in the ipsilateral CP of wild-type rats increased gradually over the period of 48 h post-TBI, with the levels of LCN2 mRNA in wild-type rats exceeding those observed in AVP-deficient rats by 19-fold.

### Vasopressin acts synergistically with TNF-α and IL-β to increase the production of proinflammatory mediators by the choroidal epithelium

**Figure 2:**
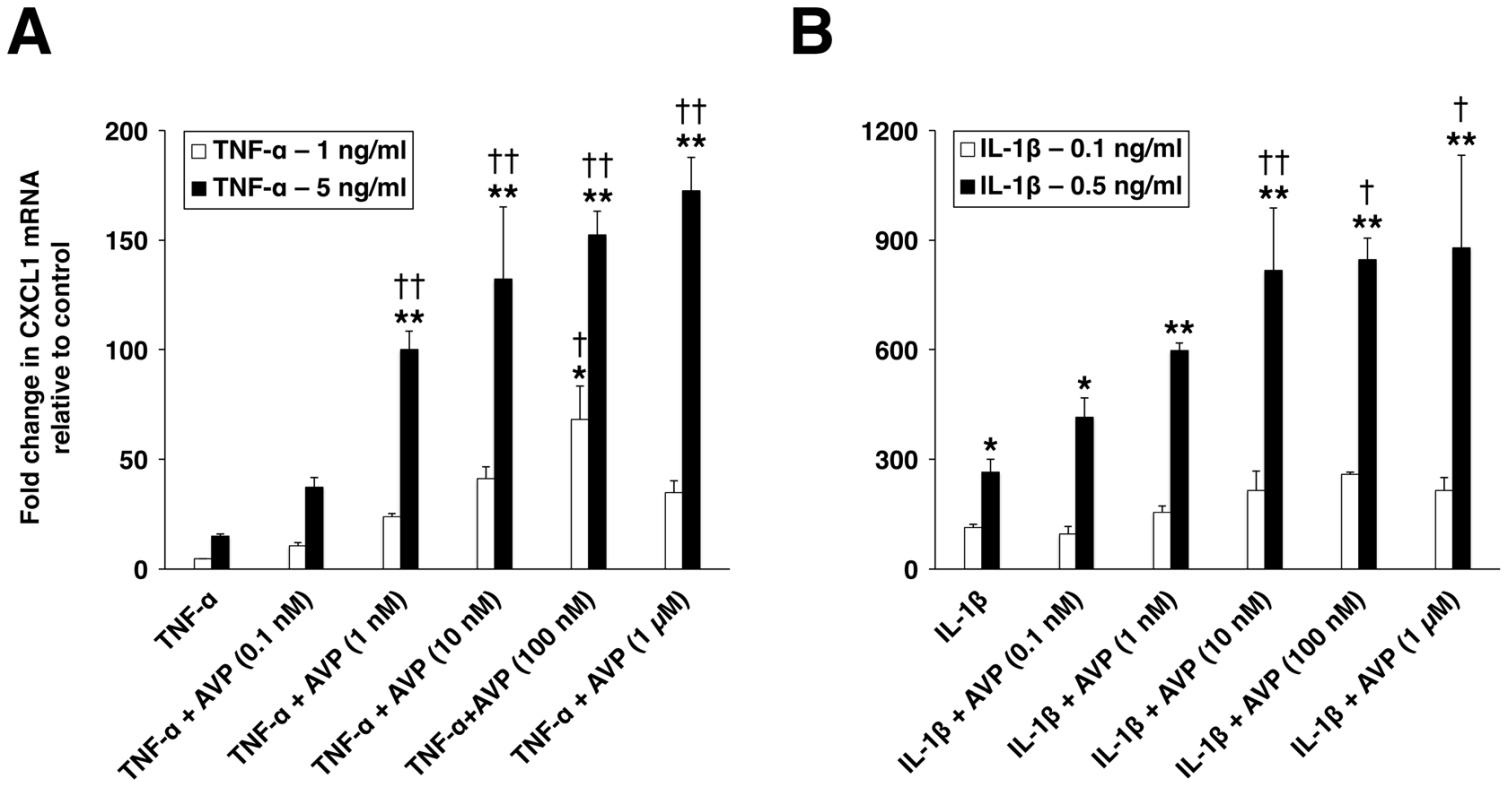
The synergistic interactions between TNF-α and AVP (A), and IL-1β and AVP (B) in the choroid plexus epithelium as assessed by real-time RT-PCR. Changes in production of CXCL1 in the choroid plexus epithelial cell line Z310 were assessed after 1-h incubation with either the cytokine (TNF-α or IL-1β) alone or a combination of the cytokine (TNF-α or IL-1β) and AVP. The exposure of Z310 cells to AVP alone at a concentration ranging between 0.1 nM and 1 µM resulted in only small increases in CXCL1 synthesis (a maximum increase observed was 6-fold relative to control with AVP at 100 nM). *p<0.05, **p<0.01 for treatment versus control. ^†^ *p*<0.05, ^††^ *p*<0.01 for TNF-α + AVP versus TNF-α alone or for IL-1β + AVP versus IL-1β alone (*n* = 3–4 per group).

We next investigated the ability of AVP to stimulate the chemokine production in the CP epithelial cell cultures. For these experiments, the CP epithelial cell line Z310 was employed. Using RT-PCR, we confirmed the presence of the AVPR1A in the Z310 cells. The AVPR1B or AVPR2 was not expressed in these cells. The effect of AVP on CXCL1 synthesis was examined, with the AVP concentration ranging between 10^–10^ and 10^–6^ M. After 1-h incubation, the maximum effect was observed at a peptide concentration of 10^–7^ M, but AVP produced only a moderate (6-fold) increase in CXCL1 mRNA. Therefore, we tested the hypothesis that AVP acts synergistically with proinflammatory cytokines to amplify the cytokine-dependent production of chemokines. A significantly larger increase in CXCL1 mRNA was observed after the Z310 cells were exposed to a combination of TNF-α and AVP compared to the increase in CXCL1 synthesis seen in these cells after exposure to TNF-α alone (Fig. 2A). Similarly, AVP augmented the IL-1β–dependent increase in expression of CXCL1, but the levels of CXCL1 mRNA were substantially higher than those found in response to TNF-α or a combination of TNF-α and AVP (Fig. 2B).

**Figure 3:**
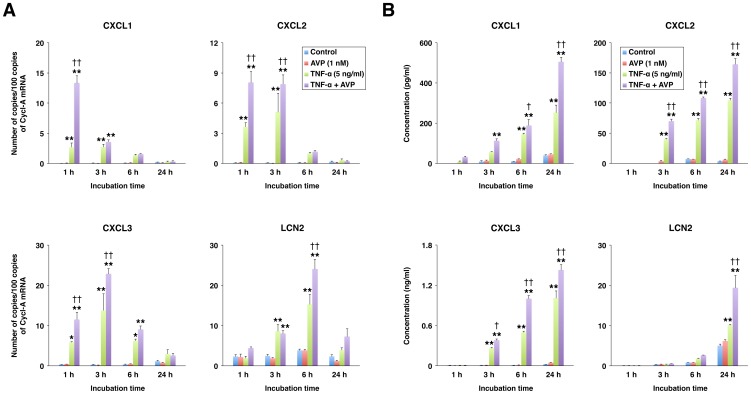
The synergistic interactions between TNF-α and AVP in the choroid plexus epithelium as assessed by real-time RT-PCR (A) and ELISA (B). The choroid plexus epithelial cells Z310 were incubated for the indicated periods of time with either TNF-α alone (5 ng/ml) or a combination of TNF-α (5 ng/ml) and AVP (1 nM). Cyclophilin A (Cycl-A) was used for the normalization of RT-PCR data. The concentrations of proinflammatory mediators were measured in the cell culture media collected at the end of the experiment. *p<0.05, **p<0.01 for treatment versus control. ^†^ *p*<0.05, ^††^ *p*<0.01 for TNF-α + AVP versus TNF-α alone (*n* = 3–4 per group).

Despite the higher levels of CXCL1 mRNA observed in response to IL-1β or a combination of IL-1β and AVP, the amplification of the TNF-α–induced CXCL1 synthesis by AVP was more robust than that of the IL-1β–induced production of this chemokine (10 times for TNF-α at 5 ng/ml versus 3 times for IL-1β at 0.5 ng/ml with AVP at 10^–7^ M). Accordingly, in subsequent experiments, the synergistic interactions between TNF-α (5 ng/ml) and AVP (10^–9^ M) were investigated. Vasopressin not only augmented the TNF-α–dependent increase in mRNA for CXCL1 but also amplified the choroidal expression of other neutrophil chemoattractants, such as CXCL2 and CXCL3, and LCN2 (Fig. 3A). Compared to other CXC chemokines studied, the production of CXCL1 was the most affected by AVP (augmented 5X by AVP), but this AVP effect was also short lasting compared to that exerted by AVP on expression of other proinflammatory mediators. The synergistic interactions between TNF-α and AVP also resulted in increased release of proinflammatory mediators into the culture media (Fig. 3B). Among the CXC chemokines studied, CXCL3 was produced at the highest rate. However, this chemokine appears to be a weaker neutrophil chemoattractant compared to CXCL2 [23], which was produced at the lowest rate.

### Vasopressin amplifies the cytokine-dependent production of proinflammatory mediators by augmenting the activation of JNK

**Figure 4:**
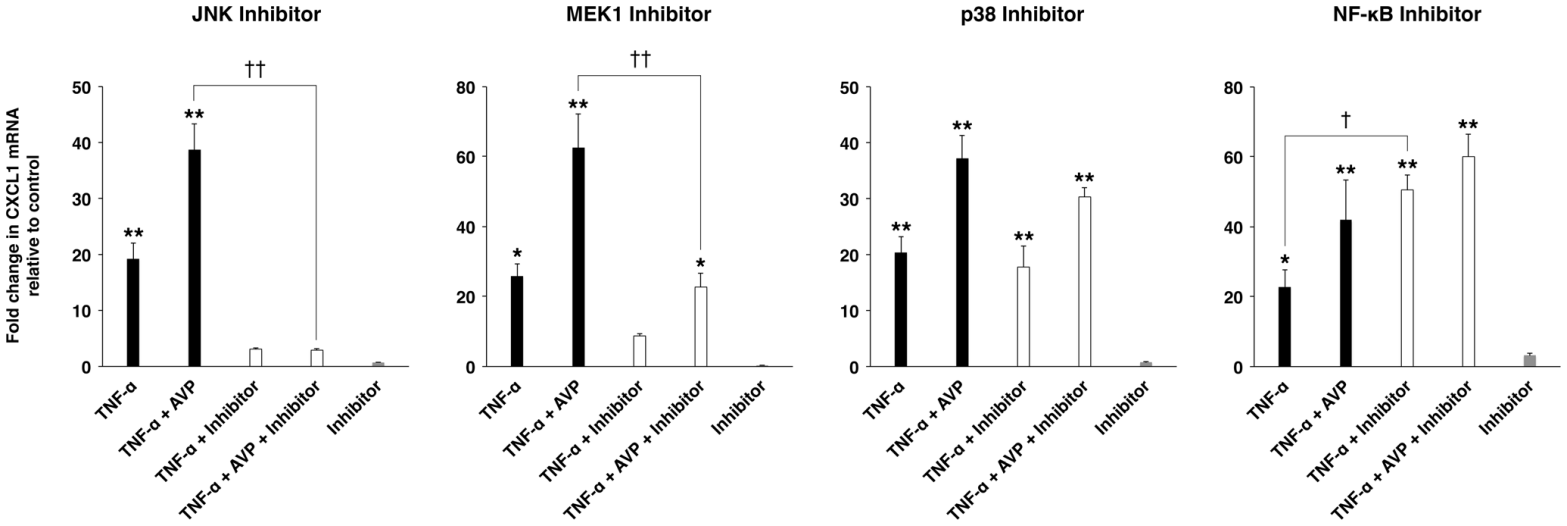
Signal transduction pathways involved in the synergistic interactions between TNF-α and AVP. The experiments were conducted on the choroid plexus epithelial cell line Z310 and changes in production of CXCL1 were assessed by real-time RT-PCR. To inhibit JNK (SP600125; 10 µM) or p38 (SB203580; 1 µM) activity, or to interfere with ERK (MEK1 inhibitor PD-98059; 10 µM) or NF-κB (SN50; 100 µg/ml) signaling, the Z3110 cells were pre-incubated for 1 h with respective inhibitors. These inhibitors were also added to the culture media when the cells were exposed to TNF-α (5 ng/ml) or a combination of TNF-α (5 ng/ml) and AVP (1 nM) for an additional hour. Note that the selective JNK inhibitor SP600125 completely abolished the induction of CXCL1 synthesis observed in response to TNF-α alone or to a combination of TNF-α and AVP. A selective MEK1 inhibitor PD-98059 partially (by >50%) inhibited CXCL1 synthesis, whereas p38 inhibitor SB203580 had no effect. In comparison, the inhibition of NF-κB signaling resulted in the amplification of chemokine synthesis. *p<0.05, **p<0.01 for treatment versus control. ^†^ *p*<0.05, ^††^ *p*<0.01 for the incubation with inhibitor versus the incubation without inhibitor (*n* = 3 per group).

To identify the signal transduction pathways mediating AVP action on the choroidal epithelium, we used selective inhibitors of JNK, MEK1, and p38. In the cultures of the Z310 cells, the inhibition of JNK completely abolished an increase in CXCL1 expression observed in response to either TNF-α or a combination of TNF-α and AVP (Fig. 4). The inhibition of ERK signaling only partially reduced the production of CXCL1, whereas the inhibition of p38 had no effect on chemokine synthesis. Interestingly, the inhibition of NF-κB signaling actually increased the synthesis of CXCL1 by the choroidal epithelium.

**Figure 5:**
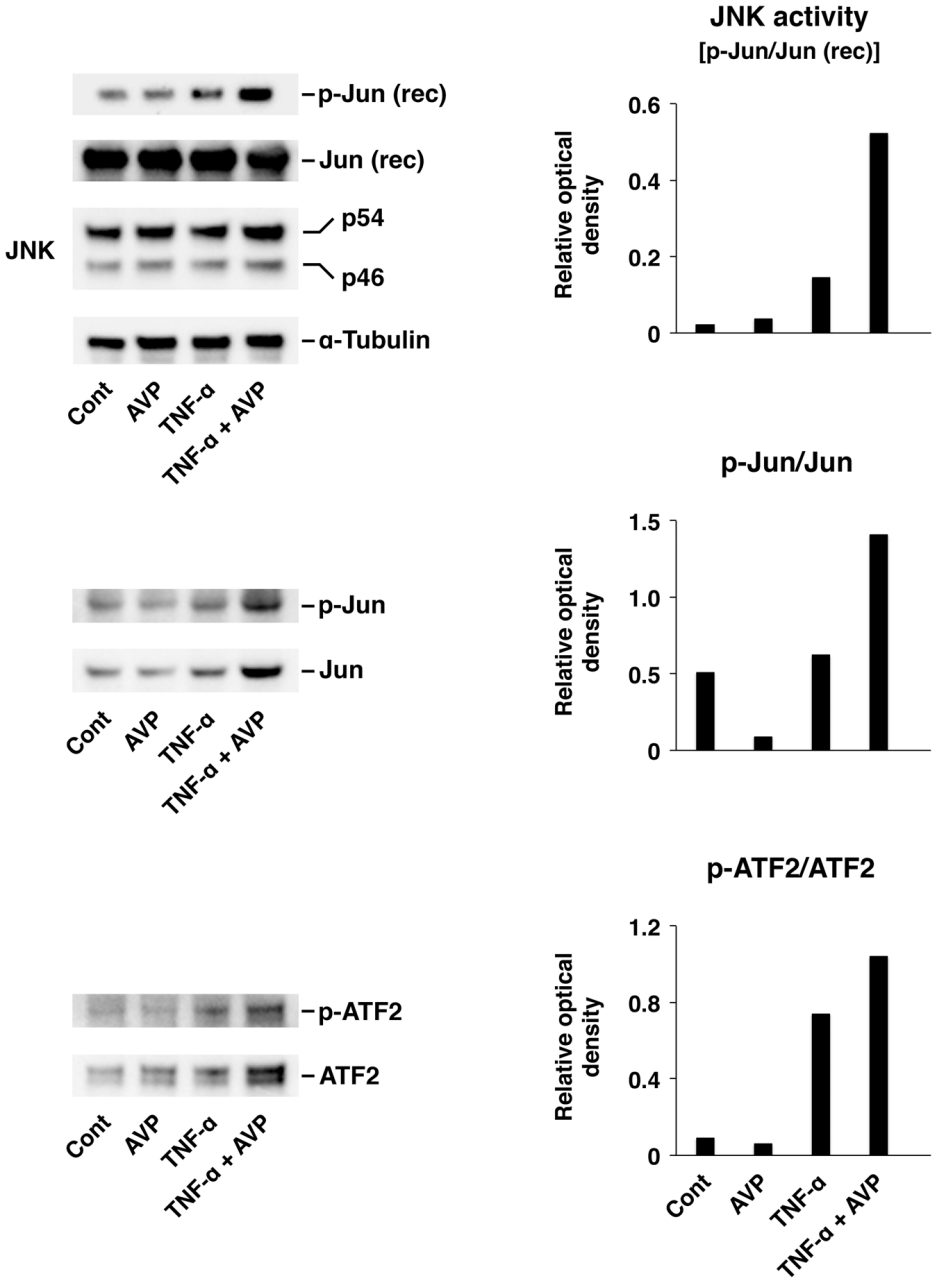
The synergistic interactions between TNF-α and AVP in the choroid plexus epithelium as assessed by the level of activation of JNK and its target transcription factors c-Jun and ATF2. The activation of the JNK signaling cascade in the Z310 cell line was assessed after 1-h incubation with either TNF-α (5 ng/ml) or a combination of TNF-α (5 ng/ml) and AVP (1 nM). In non-radioactive JNK activity assays, 200 ng of recombinant (rec) human c-Jun protein was used as a substrate for JNK. The extent of activation of c-Jun and ATF2 was assessed by Western blotting (30 µg of total protein per lane was loaded). The representative immunoblots are shown based on three independent experiments. The ratios of optical density of bands for phosphorylated proteins over the optical density of bands for the total (phosphorylated and non-phosphorylated) proteins are shown.

To confirm the results obtained with the pharmacological inhibition of JNK, we assessed changes in activation of JNK and its target transcription factors c-Jun and ATF2 in response to TNF-α or a combination of TNF-α and AVP using the JNK activity assays and Western blotting (Fig. 5). When added to the culture media at a concentration of 10^–9^ M, AVP by itself had no effect on JNK activity. However, it significantly augmented the TNF-α–dependent activation of this kinase. Similarly, AVP significantly increased the activation of c-Jun and ATF2 occurring in response to TNF-α, while on its own it had no effect on the level of phosphorylation of these transcription factors.

### Vasopressin plays an important role in augmenting the post-traumatic activation of JNK in the CP

**Figure 6:**
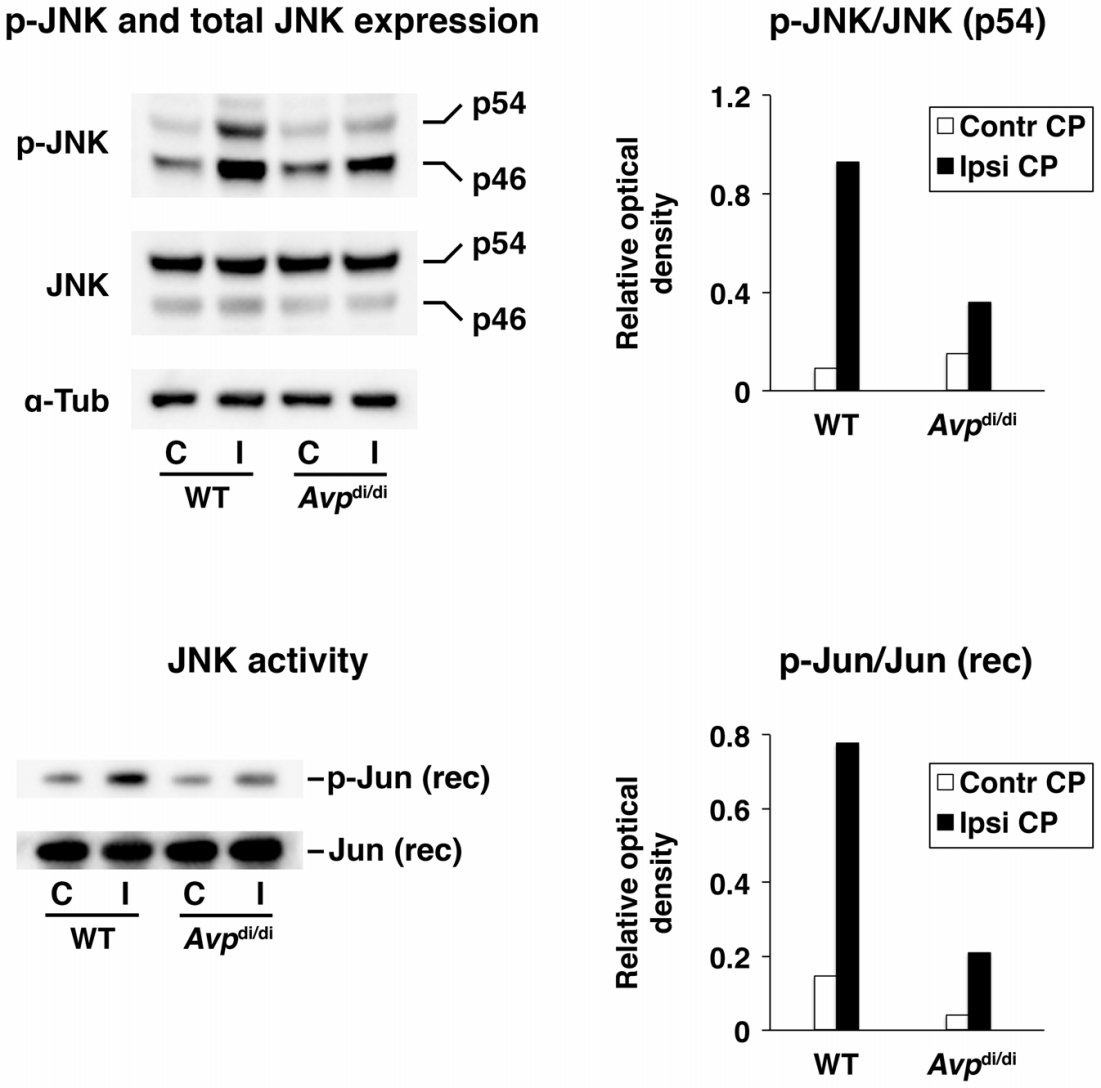
Post-traumatic activation of JNK in the lateral ventricle choroid plexus (CP) as assessed by Western blotting and JNK activity assays at 6 h after injury. A comparison of AVP-deficient Brattleboro rats (*Avp* ^di/di^) with their parental Long-Evans strain (WT). The activity of JNK was assessed in both the ipsilateral (Ipsi CP/I) and contralateral (Contr CP/C) CPs. For Western blotting, 40 µg of total protein per lane was loaded. In non-radioactive JNK activity assays, 200 ng of recombinant (rec) human c-Jun protein was used as a substrate for JNK. Similar results were obtained in an independent experiment involving separate groups of animals. The ratios of optical density of bands for phosphorylated proteins over the optical density of bands for the total (phosphorylated and non-phosphorylated) proteins are shown (*n* = 4 per group).

In wild-type rats, TBI resulted in a rapid (within 6 h post-injury) activation of JNK in the ipsilateral CP, which was determined by Western blotting with phospho-specific anti-JNK antibodies and the JNK activity assays (Fig. 6). The extent of post-traumatic activation of JNK in the ipsilateral CP was considerably attenuated in *Avp* ^di/di^ rats, suggesting the important role of this neuropeptide in the activation of this signaling pathway after injury.

### In vivo inhibition of JNK decreases the post-traumatic production of neutrophil chemoattractants by the CP and reduces the influx of neutrophils across the BCSFB

**Figure 7:**
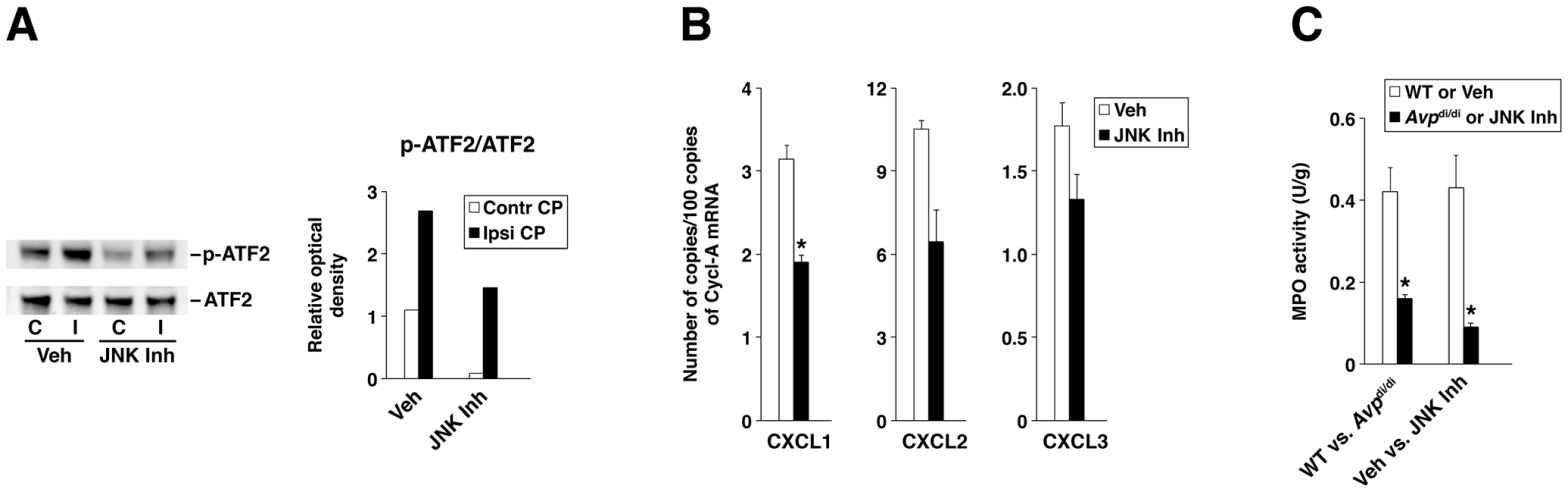
The effect of inhibition of JNK in vivo. To inhibit the activity of JNK in rats sustaining TBI, a selective JNK inhibitor SP600125 (JNK Inh) was injected i.p. at 4 and 8 h post-TBI at a dose of 30 mg/kg for each injection. A control group of rats was injected with vehicle (Veh; DMSO). (A) The efficacy of pharmacological inhibition of JNK as assessed by the reduction in post-traumatic activation of ATF2 in the lateral ventricle choroid plexus (CP) at 6 h after injury. Another target transcription factor for JNK, c-Jun, was undetectable in the CP. The activity of ATF2 was assessed by Western blotting in both the ipsilateral (Ipsi CP/I) and contralateral (Contr CP/C) CPs (30 µg of total protein per lane was loaded). The representative immunoblots are shown based on two independent experiments involving separate groups of animals. The ratios of optical density of bands for phosphorylated ATF2 over the optical density of bands for the total (phosphorylated and non-phosphorylated) ATF2 are shown. (B) The effect of JNK inhibition on post-traumatic production of neutrophil chemoattractants CXCL1–3 by the lateral ventricle CP as assessed by real-time RT-PCR at 6 h after injury. Cyclophilin A (Cycl-A) was used for the normalization of the data. (C) The role of AVP in promoting the post-traumatic influx of neutrophils across the blood-cerebrospinal fluid barrier (BCSFB) and the effect of JNK inhibition on this influx. To define the pathophysiological role of AVP in promoting neutrophil influx across the BCSFB, AVP-deficient Brattleboro rats (*Avp* ^di/di^) were compared with their parental Long-Evans strain (WT). The magnitude of neutrophil influx was assessed by the myeloperoxidase (MPO) activity assays at 24 h post-TBI. *p<0.05 for JNK Inh versus Veh or for *Avp* ^di/di^ versus WT (*n* = 4–6 per group).

To inhibit the activity of JNK in wild-type rats sustaining injury, a selective JNK inhibitor SP600125 was administered at 4 and 8 h post-TBI. The efficacy of this pharmacological inhibition of JNK was judged by the extent of reduction in post-traumatic activation of ATF2 in the ipsilateral CP. Another target transcription factor for JNK, c-Jun, was undetectable in the CP. The administration of SP600125 resulted in a significant reduction in the level of post-traumatic activation of ATF2 in the ipsilateral CP when assessed at 6 h after injury (Fig. 7A). Consequently, a decrease in post-traumatic production of neutrophil chemoattractants in the ipsilateral CP was also observed (Fig. 7B). To determine the effect of JNK inhibition on the magnitude of post-traumatic influx of neutrophils across the BCSFB, we employed the MPO assays. The influx of neutrophils was assessed at 24 h post-TBI, a time point after injury at which the maximum accumulation of these inflammatory cells in the ipsilateral CP was seen [4]. Consistent with the reduction in post-traumatic production of neutrophil chemoattractants observed after the pharmacological inhibition of JNK, we also found a reduction (by 79%) in the magnitude of neutrophil influx across the BCSFB in animals treated with SP600125 versus those receiving vehicle (Fig. 7C). When we compared wild-type rats with AVP-deficient animals, we also observed a significant reduction (by 62%) in the magnitude of post-traumatic influx of neutrophils across the BCSFB (Fig. 7C). These observations support an important role of AVP in augmenting the post-traumatic invasion of neutrophils across the BCSFB.

## Discussion

In this study, we have demonstrated the synergistic interactions between proinflammatory cytokines and AVP, a ligand for G protein-coupled receptors. By interacting with TNF-α and IL-1β, AVP amplifies the cytokine-dependent production of proinflammatory mediators by the CP, consequently exacerbating the brain inflammatory response to injury. This AVP action is mediated by its cognate AVPR1A receptors. We have confirmed by RT-PCR that the AVPR1B or AVPR2 is not expressed in the CP. Our observations challenge the traditional view of AVP as a pressor/antidiuretic hormone and support a new role for AVP as a regulator of post-traumatic neuroinflammation. Clinical studies have shown that TBI increases the levels of circulating AVP [24]. There is also evidence based on studies of experimental TBI that brain injury results in a rapid increase in AVP concentration in the CSF [25]. We have previously shown that the AVPR1A is localized to the apical (CSF-facing) surface of the choroidal epithelium [17]. This suggests that AVP released into the CSF after injury augments the post-traumatic production of proinflammatory mediators by the CP.

When compared with AVP-deficient rats, wild-type animals had increased synthesis of neutrophil chemoattractants in the ipsilateral CP after neurotrauma. This was associated with the larger magnitude of post-traumatic influx of neutrophils across the BCSFB in wild-type rats compared to *Avp* ^di/di^ rats. In both *in vivo* and *in vitro* studies, a short-lasting (for up to 6 h) increase in mRNA for neutrophil chemoattractants was only observed. However, studies in the Z310 cells demonstrated a prolonged (lasting for at least 24 h) increase in production of chemokine proteins, with AVP having the most significant effect on TNF-α–dependent chemokine synthesis at later time points during the 24-h experimental period. This may explain why a peak in the magnitude of post-traumatic influx of neutrophils across the BCSFB is observed at 24 h after injury, and a very limited accumulation of these inflammatory cells in the ipsilateral CP is seen at 6–8 h post-TBI [4].

The synergistic interactions between proinflammatory cytokines and AVP are predominantly mediated by the JNK signaling pathway. *In vivo* and *in vitro* studies indicated that AVP by itself has little effect on JNK activity in the CP. However, AVP could effectively enhance JNK activation induced by cytokines and, possibly, other factors involved in the initiation and propagation of inflammatory cascade in the injured brain. Previous studies have demonstrated that the JNK signaling pathway plays a significant part in regulating the synthesis of neutrophil and monocyte chemoattractants [26]–[28], which supports the mediatory role of JNK in AVP action. Interestingly, we have found that NF-κB plays a negative regulatory role in AVP-dependent amplification of choroidal synthesis of chemokines. When the selective NF-κB inhibitor was added to the Z310 cell culture media, a larger increase in chemokine synthesis in response to TNF-α or a combination of TNF-α and AVP was observed. Our findings are consistent with previous observations that NF-κB deficiency prolongs the TNF-α–dependent activation of JNK [29]. These previous studies have also demonstrated that TNF-α induces the production of reactive oxygen species (ROS) that inhibit JNK-inactivating phosphatases, and NF-κB acts to reduce the TNF-α–dependent production of ROS. There is evidence that AVP has also the ability to induce the production of ROS [30]. It is thus possible that AVP augments the cytokine-dependent synthesis of proinflammatory mediators by inhibiting JNK-inactivating phosphatases.

## References

[1] Strazielle N, Ghersi-Egea JF (2000) Choroid plexus in the central nervous system: biology and physiopathology. J Neuropathol Exp Neurol 59: 561–574. 10901227 10.1093/jnen/59.7.561

[2] Reboldi A, Coisne C, Baumjohann D, Benvenuto F, Bottinelli D, et al (2009) C-C chemokine receptor 6-regulated entry of TH-17 cells into the CNS through the choroid plexus is required for the initiation of EAE. Nat Immunol 10: 514–523. 19305396 10.1038/ni.1716

[3] Schmitt C, Strazielle N, Ghersi-Egea JF (2012) Brain leukocyte infiltration initiated by peripheral inflammation or experimental autoimmune encephalomyelitis occurs through pathways connected to the CSF-filled compartments of the forebrain and midbrain. J Neuroinflammation 9: 187. 22870891 10.1186/1742-2094-9-187 PMC3458946

[4] Szmydynger-Chodobska J, Strazielle N, Zink BJ, Ghersi-Egea JF, Chodobski A (2009) The role of the choroid plexus in neutrophil invasion after traumatic brain injury. J Cereb Blood Flow Metab 29: 1503–1516. 19471279 10.1038/jcbfm.2009.71 PMC2736364

[5] Szmydynger-Chodobska J, Strazielle N, Gandy JR, Keefe TH, Zink BJ, et al (2012) Posttraumatic invasion of monocytes across the blood-cerebrospinal fluid barrier. J Cereb Blood Flow Metab 32: 93–104. 21829211 10.1038/jcbfm.2011.111 PMC3323293

[6] Utagawa A, Bramlett HM, Daniels L, Lotocki G, Dekaban GA, et al (2008) Transient blockage of the CD11d/CD18 integrin reduces contusion volume and macrophage infiltration after traumatic brain injury in rats. Brain Res 1207: 155–163. 18374312 10.1016/j.brainres.2008.02.057 PMC2435262

[7] Bao F, Shultz SR, Hepburn JD, Omana V, Weaver LC, et al (2012) A CD11d monoclonal antibody treatment reduces tissue injury and improves neurological outcome after fluid percussion brain injury in rats. J Neurotrauma 29: 2375–2392. 22676851 10.1089/neu.2012.2408 PMC4854615

[8] Semple BD, Bye N, Ziebell JM, Morganti-Kossmann MC (2010) Deficiency of the chemokine receptor CXCR2 attenuates neutrophil infiltration and cortical damage following closed head injury. Neurobiol Dis 40: 394–403. 20621186 10.1016/j.nbd.2010.06.015

[9] Kenne E, Erlandsson A, Lindbom L, Hillered L, Clausen F (2012) Neutrophil depletion reduces edema formation and tissue loss following traumatic brain injury in mice. J Neuroinflammation 9: 17. 22269349 10.1186/1742-2094-9-17 PMC3292978

[10] Semple BD, Bye N, Rancan M, Ziebell JM, Morganti-Kossmann MC (2010) Role of CCL2 (MCP-1) in traumatic brain injury (TBI): evidence from severe TBI patients and CCL2–/– mice. J Cereb Blood Flow Metab 30: 769–782. 20029451 10.1038/jcbfm.2009.262 PMC2949175

[11] Chodobski A, Zink BJ, Szmydynger-Chodobska J (2013) CNS barriers in neurotrauma. In: Lo EH, Lok JM, Whalen M, editors.Vascular Mechanisms in CNS Trauma. Springer Science (in press).

[12] Trabold R, Krieg S, Scholler K, Plesnila N (2008) Role of vasopressin V_1a_ and V_2_ receptors for the development of secondary brain damage after traumatic brain injury in mice. J Neurotrauma 25: 1459–1465. 19118456 10.1089/neu.2008.0597

[13] Kleindienst A, Dunbar JG, Glisson R, Marmarou A (2013) The role of vasopressin V_1A_ receptors in cytotoxic brain edema formation following brain injury. Acta Neurochir (Wien) 155: 151–164. 23188468 10.1007/s00701-012-1558-z

[14] Rauen K, Trabold R, Brem C, Terpolilli NA, Plesnila N (2013) Arginine vasopressin V_1a_ receptor-deficient mice have reduced brain edema and secondary brain damage following traumatic brain injury. J Neurotrauma 30: 1442–1448. 23441636 10.1089/neu.2012.2807

[15] Vakili A, Kataoka H, Plesnila N (2005) Role of arginine vasopressin V_1_ and V_2_ receptors for brain damage after transient focal cerebral ischemia. J Cereb Blood Flow Metab 25: 1012–1019. 15744246 10.1038/sj.jcbfm.9600097

[16] Manaenko A, Fathali N, Khatibi NH, Lekic T, Hasegawa Y, et al (2011) Arginine-vasopressin V_1a_ receptor inhibition improves neurologic outcomes following an intracerebral hemorrhagic brain injury. Neurochem Int 58: 542–548. 21256175 10.1016/j.neuint.2011.01.018 PMC3063401

[17] Szmydynger-Chodobska J, Chung I, Koźniewska E, Tran B, Harrington FJ, et al (2004) Increased expression of vasopressin V_1a_ receptors after traumatic brain injury. J Neurotrauma 21: 1090–1102. 15319008 10.1089/0897715041651033

[18] Szmydynger-Chodobska J, Chung I, Chodobski A (2006) Chronic hypernatremia increases the expression of vasopressin and voltage-gated Na channels in the rat choroid plexus. Neuroendocrinology 84: 339–345. 17164538 10.1159/000097989

[19] Schmale H, Richter D (1984) Single base deletion in the vasopressin gene is the cause of diabetes insipidus in Brattleboro rats. Nature 308: 705–709. 6717565 10.1038/308705a0

[20] Zheng W, Zhao Q (2002) Establishment and characterization of an immortalized Z310 choroidal epithelial cell line from murine choroid plexus. Brain Res 958: 371–380. 12470873 10.1016/s0006-8993(02)03683-1 PMC3980880

[21] Szmydynger-Chodobska J, Pascale CL, Pfeffer AN, Coulter C, Chodobski A (2007) Expression of junctional proteins in choroid plexus epithelial cell lines: a comparative study. Cerebrospinal Fluid Res 4: 11. 18162136 10.1186/1743-8454-4-11 PMC2241822

[22] Marques F, Rodrigues AJ, Sousa JC, Coppola G, Geschwind DH, et al (2008) Lipocalin 2 is a choroid plexus acute-phase protein. J Cereb Blood Flow Metab. 28: 450–455. 17895910 10.1038/sj.jcbfm.9600557

[23] Nakagawa H, Komorita N, Shibata F, Ikesue A, Konishi K, et al (1994) Identification of cytokine-induced neutrophil chemoattractants (CINC), rat GRO/CINC-2α and CINC-2β, produced by granulation tissue in culture: purification, complete amino acid sequences and characterization. Biochem J 301: 545–550. 8043001 10.1042/bj3010545 PMC1137115

[24] Xu M, Su W, Huang WD, Lu YQ, Xu QP, et al (2007) Effect of AVP on brain edema following traumatic brain injury. Chin J Traumatol 10: 90–93. 17371619

[25] Armstead WM (1996) Role of vasopressin in altered pial artery responses to dynorphin and β-endorphin following brain injury. J Neurotrauma 13: 115–123. 8965321 10.1089/neu.1996.13.115

[26] Kim DS, Han JH, Kwon HJ (2003) NF-κB and c-Jun-dependent regulation of macrophage inflammatory protein-2 gene expression in response to lipopolysaccharide in RAW 264.7 cells. Mol Immunol 40: 633–643. 14597166 10.1016/j.molimm.2003.07.001

[27] Wang Y, Luo W, Stricker R, Reiser G (2006) Protease-activated receptor-1 protects rat astrocytes from apoptotic cell death via JNK-mediated release of the chemokine GRO/CINC-1. J Neurochem 98: 1046–1060. 16749907 10.1111/j.1471-4159.2006.03950.x

[28] Hanazawa S, Takeshita A, Amano S, Semba T, Nirazuka T, et al (1993) Tumor necrosis factor-α induces expression of monocyte chemoattractant JE via *fos* and *jun* genes in clonal osteoblastic MC3T3-E1 cells. J Biol Chem 268: 9526–9532. 8486642

[29] Kamata H, Honda S, Maeda S, Chang L, Hirata H, et al (2005) Reactive oxygen species promote TNFα-induced death and sustained JNK activation by inhibiting MAP kinase phosphatases. Cell 120: 649–661. 15766528 10.1016/j.cell.2004.12.041

[30] Armstead WM (2001) Vasopressin-induced protein kinase C-dependent superoxide generation contributes to ATP-sensitive potassium channel but not calcium-sensitive potassium channel function impairment after brain injury. Stroke 32: 1408–1414. 11387506 10.1161/01.str.32.6.1408

